# Double pituitary adenomas: report of two cases and systematic review of the literature

**DOI:** 10.3389/fendo.2024.1373869

**Published:** 2024-04-02

**Authors:** Yi Zhang, Xinyue Gong, Jun Pu, Jifang Liu, Zhang Ye, Huijuan Zhu, Lin Lu, Hui Pan, Kan Deng, Yong Yao

**Affiliations:** ^1^ Department of Neurosurgery, Peking Union Medical College Hospital, Chinese Academy of Medical Science and Peking Union Medical College, Beijing, China; ^2^ Eight-Year Program of Clinical Medicine, Peking Union Medical College, Chinese Academy of Medical Science & Peking Union Medical College, Beijing, China; ^3^ Department of Endocrinology, Peking Union Medical College Hospital, Chinese Academy of Medical Science and Peking Union Medical College, Beijing, China

**Keywords:** double pituitary adenomas (DPA), cushing disease, acromegaly, biochemical remission, prognostic analysis

## Abstract

**Objective:**

Double pituitary adenomas (DPA) are a rare clinical condition, and our knowledge of them is limited. Missing the second lesion leading to incomplete biochemical remission after surgery is an important challenge in DPA management. This study aims to analyze independent prognostic factors in DPA patients and summarize clinical experiences to prevent surgical failure.

**Methods:**

Two cases of DPA patients with Cushing’s disease diagnosed and surgically treated at Peking Union Medical College Hospital are reported. A literature review was performed on the online database Pubmed, and 57 DPA patients from 22 retrieved articles were included. Demographic characteristics, endocrine manifestations, diagnostic methods, tumor size, and immunohistochemical features of 59 patients were analyzed. Binary logistic regression models were used to identify independent prognostic factors affecting postoperative biochemical remission.

**Results:**

Among 59 DPA patients, the mean ± SD age was 43.64 ± 14.42 years, with 61.02% being female (n = 36). The most common endocrine manifestations were Cushing’s syndrome (23/59, 38.98%) and acromegaly (20/59, 33.90%). The most prevalent immunohistochemical types were ACTH-immunopositive (31/118, 26.27%) and GH-immunopositive (31/118, 26.27%) tumors. Microadenomas (<1cm) were the most frequent in terms of tumor size (62/92, 67.39%). The detection rate for double lesions on 3.0T MRI was 50.00% (14/28), which significantly higher than 1.5T MRI (P = 0.034). Univariate analysis revealed that female, Cushing’s syndrome and only single lesion detected by surgical exploration were associated with significantly worse prognosis (P<0.05). Multivariate analysis identified double lesion detected by surgical exploration (OR = 0.08, P = 0.003) and contiguous type tumor (OR = 0.06, P = 0.017) as independent protective factors for DPA patients.

**Conclusions:**

The double lesion detected by surgical exploration is independently associated with a better prognosis for DPA patients. Comprehensive intraoperative exploration are crucial measures to avoid missing causative lesions.

## Introduction

Pituitary adenoma is the third most common intracranial tumor, following glioma and meningioma, accounting for about 15% of intracranial tumors (1, 2). Pituitary adenomas are the predominant pathological type of masses in the sellar and suprasellar region, accounting for up to 90% of cases (3). Clinically, pituitary adenomas present with endocrine symptoms associated with inappropriate pituitary hormone secretion as well as local mass effects. Typically benign, monoclonal, and slow-growing solid tumors, pituitary adenomas rarely exhibit invasive or malignant behavior (4). The estimated incidence of pituitary adenomas is approximately 25 cases per 100,000 individuals (5), with the vast majority of patients having a solitary tumor. The occurrence of two pituitary adenomas in one single patient is extremely low, with a prevalence of 0.2-2.6% among pituitary adenoma surgical cohorts (6).

Double pituitary adenoma (DPA) is defined as the synchronous presence of two distinct pituitary adenomas in the pituitary gland, exhibiting differences in morphology, immunohistochemistry, and ultrastructural features. They can be either two separate masses that exist independently of each other in the pituitary gland (separated type) or a single gross lesion with a histologically well-delineated border to distinguish two distinct cell populations (contiguous type). Notably, the concept of DPA should be distinguished from a plurihormonal adenoma, which is a single tumor capable of secreting two or more pituitary hormones simultaneously, composed of one homogeneous and histologically-uniform tumor cell population. The clinical management of patients with DPA presents significant challenges, including low imaging detection rates leading to diagnostic difficulties, challenges in achieving complete resection of pituitary adenoma tissue during surgery, and the potential risk for secondary surgery due to postoperative biochemical non-remission. Therefore, paying more attention to this rare clinical scenario is necessary.

Although there are review articles for DPA, to our knowledge, few authors have summarized this uncommon disease in a systematic way, especially the lack of prognostic analysis of clinical outcomes. In this article, we reported 2 patients with DPA presenting as Cushing’s syndrome and selected 57 cases of functional DPA from the previous literature. Finally, we conducted a systematic review of 59 functional DPA, summarizing their demographics, clinical features, and tumor characteristics, analyzing prognostic factors related to postoperative biochemical remission, and providing insights into diagnostic and therapeutic management practices for patients with DPA.

## Cases presentation

### Case 1

A 35-year-old female patient was admitted to the hospital due to a two-year history of facial roundness and redness. She exhibits the clinical features of classic hypercortisolism, including unexplained significant weight gain, acne on the face and back, and thinning of the skin with bruising. Symptoms exhibited seasonal fluctuations, with more severe manifestations in winter. Physical examination revealed signs of Cushing’s syndrome, including central obesity, moon face, buffalo hump, plethora, supraclavicular fat pad, increased vellus hair, and abdominal striae.

Biochemical results are shown in **Table 1**. Biochemical examination revealed elevated serum cortisol (21.9 μg/dL) and adrenocorticotrophic hormone (ACTH) levels (58.5 pg/mL) with the disappearance of circadian rhythm. The low-dose dexamethasone suppression test (LDDST) showed incomplete suppression of both blood cortisol and 24-hour urinary free cortisol (24h UFC). Continuous monitoring of 24h UFC revealed significant fluctuations (range: 347.7-992.3 μg/24h) and a “triple peak and double trough” pattern, confirming cyclic Cushing’s syndrome.

**Table 1:** Assessment of anterior pituitary function before and after surgery.

Hormone	Case 1, F		Case 2, M		Normal range	
	Before	After	Before	After		
ACTH (0 AM), pg/mL	54.8	—	—	—	7.2-63.3	
ACTH (8 AM), pg/mL	58.5	6.5	129.0	14.3	7.2-63.3	
Cortisol (0 AM), μg/dL	18.9	—	21.7	—	4.0-22.3	
Cortisol (8 AM), μg/dL	21.9	1.2	26.9	4.2	4.0-22.3	
24h UFC, μg/24h	229.2	42.9	199.5	25.2	13.2-77.2	
GH, ng/mL	1.5	1.8	0.1	0.3	<2.0	
IGF-1, ng/mL	271	320	176	199	63-223	
TSH3, μIU/mL	0.390	0.284	1.568	0.206	0.380-4.340	
FT3, pg/mL	2.62	2.90	2.99	2.27	1.80-4.10	
FT4, ng/mL	1.07	1.75	1.35	1.30	0.81-1.89	
					For female	For male
LH, IU/mL	7.16	1.2	3.49	1.25	2.12-10.89	1.24-6.62
FSH, IU/mL	4.20	2.04	6.25	4.89	<10.00	1.27-19.26
E2, pg/mL	86	35	22	< 15	22-115	< 39
P, ng/mL	0.34	1.25	0.30	1.52	0.38-2.28	0.10-0.84
T, ng/mL	0.64	<0.1	1.70	0.89	0.10-0.84	1.75-7.81
PRL, ng/mL	16.9	1.2	12.1	0.9	<30.0	2.6-13.1

ACTH, adrenocorticotrophic hormone; E2, estradiol; FSH, follicle-stimulating hormone; FT3, free triiodothyronine; FT4, free thyronine; GH, growth hormone; IGF-1, insulin-like growth factor 1; LH, luteinizing hormone; P, progesterone; PRL, prolactin; T, testosterone; TSH, thyroid-stimulating hormone; 24h UFC, 24h urine free cortisol.

**Figure 1:**
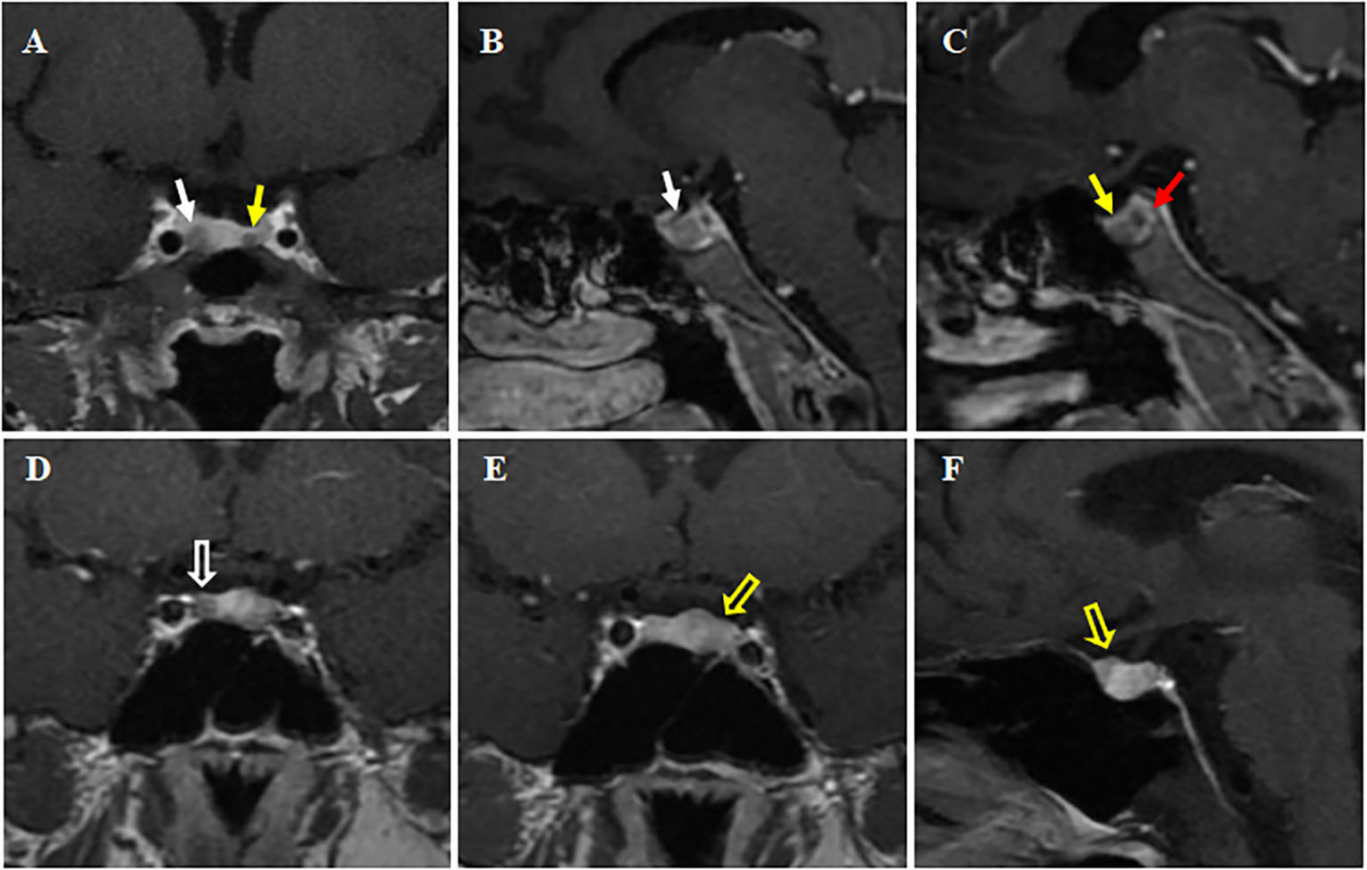
Preoperative dynamic contrast-enhanced MRI of the pituitary. **(A-C)** Case 1. The pituitary gland demonstrates heterogeneous enhancement, with bilateral areas of hypointense signal. Larger (about 11.3 × 5.5 mm) on right side (white solid arrows, **(A, B)**; Smaller (about 3 × 3.5 mm) on left side (yellow solid arrow, **A, C**). A red solid arrow points to a non-enhancing area, representing a posterior pituitary cyst **(C)**. **(D-F)** Case 2. A rounded hypo-enhanced signal about 5 mm in diameter is seen on the right side (white hollow arrow, in **D**), and a patchy area of reduced enhancement is observed on the left anterior side (yellow hollow arrows, in **E, F**). All A-F are post-contrast T1-weighted sequences. Coronal views in **(A, D, E)**; Sagittal views in **(B, C, F)**.

The high-dose dexamethasone suppression test (HDDST) completely suppressed serum cortisol and 24h UFC levels, supporting the diagnosis of Cushing’s disease. Dynamic contrast-enhanced MRI of the pituitary showed hypo-enhanced signal intensity on both sides of the pituitary gland, about 11.3 × 5.5 mm on the right side and 3 × 3.5 mm on the left side, which was considered as double pituitary adenomas (**Figures 1A–C**). Bilateral inferior petrosal sinus sampling (BIPSS) supported the left side as the dominant ACTH-secreting side.

Preoperative evaluation showed normal anterior pituitary function except for the adrenal axis, and Cushing’s syndrome complications were managed symptomatically. In addition, we noticed the mild elevation of IGF-1 despite normal GH and performed glucose suppression test, which showed that GH could be completely suppressed and did not support a GH-secreting adenoma. The elevated IGH-1 was considered to be associated with long-term hypercortisolism. Ophthalmologic evaluation, including visual acuity and dynamic visual field test, revealed no abnormalities.

**Figure 2:**
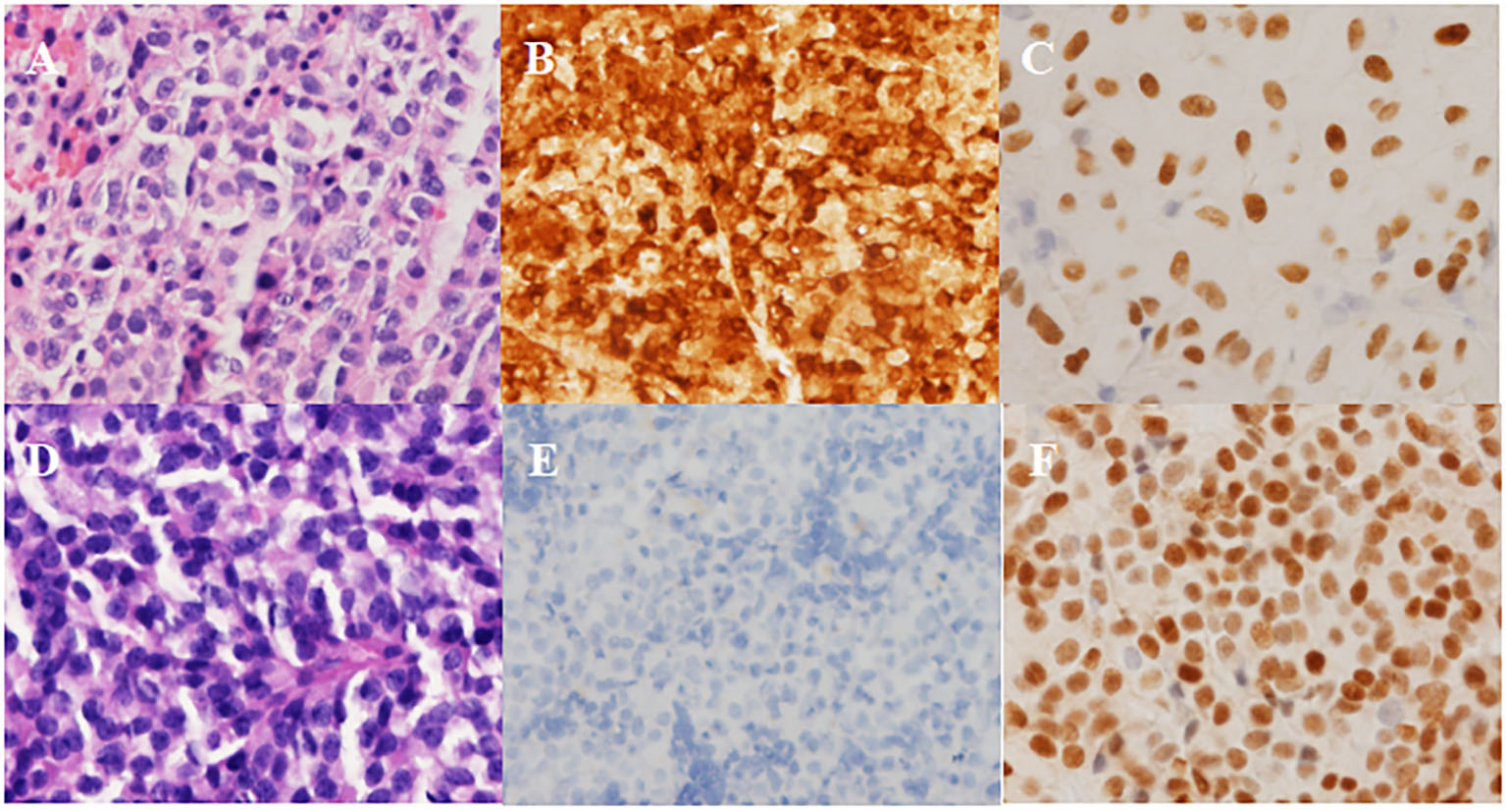
Pathological results in Case 1. **(A-C)** Left-side adenoma: strongly positive staining for ACTH and positive for T-pit. **(A)** H&E, **(B)** ICH for ACTH, **(C)** ICH for T-pit. **(D-F)** Right-side adenoma: negative staining for ACTH and positive for SF-1. **(D)** H&E, **(E)** ICH for ACTH, **(F)** ICH for SF-1. Magnification × 200.

The patient underwent sellar lesions resection and sellar floor reconstruction through the neuroendoscopic endonasal transsphenoidal approach. Two soft gray-white tumors were found on the right and left sides of the intrasellar region and completely removed during the operation. A thorough endoscopic exploration of the sellar region was performed. Immunohistochemical examination confirmed strong positive ACTH staining and the expression of T-pit transcription factor in the left-sided adenoma, while the right-sided adenoma showed negative staining for any pituitary hormones and expressed the SF-1 transcription factor (**Figure 2**).

On day 2 after the operation, a follow-up assessment of anterior pituitary function was conducted, and the patient achieved complete biochemical remission with significant decreases in serum ACTH, cortisol, and 24h UFC levels. Postoperative oral hydrocortisone replacement therapy was given because of hypopituitarism.

### Case 2

A 35-year-old man was admitted to the hospital with complaints of unexplained weight gain for 4 years, with a weight increase of 25 kg in the last year. Three years ago, he noticed facial rounding and redness, abdominal striae, skin prone to bruising, and elevated blood pressure reaching a maximum of 180/120 mmHg. In the last 3 months, he experienced polydipsia, polyuria, lower limb and eyelid edema, and reduced sexual function, and was diagnosed as diabetes mellitus and hyperlipidemia. Physical examination demonstrated typical signs of Cushing’s syndrome, including central obesity, moon face, buffalo hump, thickened fat pad above the clavicles, abdominal and thigh striae, and limb bruising.

Biochemical tests revealed a loss of circadian rhythm in cortisol, which was as high as 26.9 μg/dL at 8 AM, with an elevated 24-h UFC of 199.5 μg/24 h and an elevated ACTH of 129.0 pg/mL. LDDST revealed non-suppressed cortisol levels (26.4 μg/dL). BIPSS supported a diagnosis of Cushing’s disease with left-side dominance. Dynamic contrast-enhanced pituitary MRI revealed a reduced enhancing area on the anterior left wing of the pituitary measuring approximately 5 × 3.5 mm, and another reduced enhancing area about 5mm in diameter on the right wing, suggesting the presence of double pituitary adenomas (**Figures 1D–F**).

Preoperative evaluation of other pituitary hormones showed no significant abnormality. Ophthalmological evaluation ruled out visual impairment and visual field defects.

**Figure 3:**
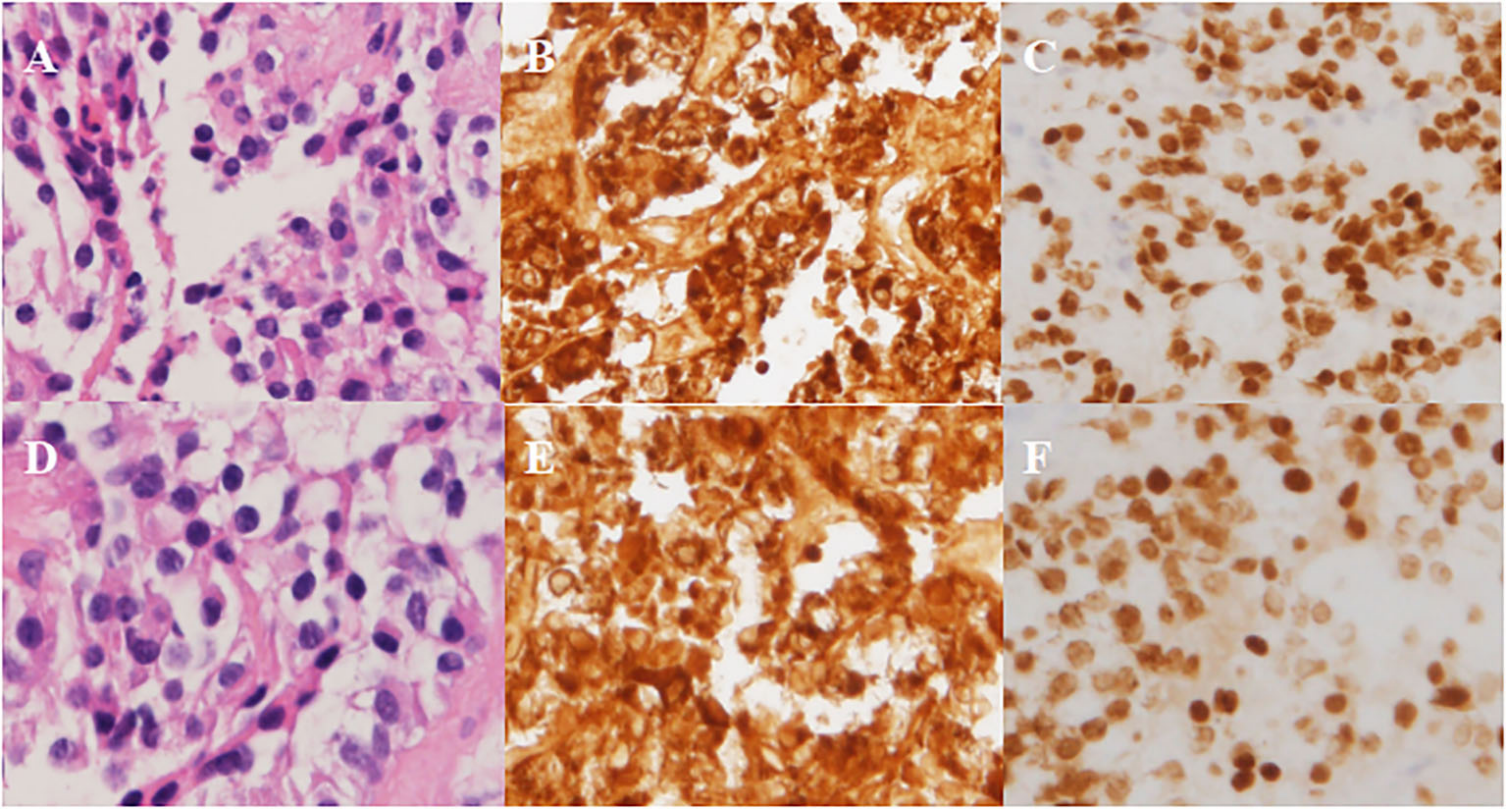
Pathological results in Case 2. **(A-C)** Left-side adenoma: positive staining for ACTH and T-pit. **(A)** H&E, **(B)** ICH for ACTH, **(C)** ICH for T-pit. **(D-F)** Right-side adenoma: positive staining for ACTH and T-pit. **(D)** H&E, **(E)** ICH for ACTH, **(F)** ICH for T-pit. Magnification × 200.

The patient underwent sellar lesions resection and sellar floor reconstruction through neuroendoscopic endonasal transsphenoidal approach. Intraoperative pituitary adenomas on both sides were explored and found, completely excised and sent for pathologic examination separately. The immunohistochemical results confirmed that both pituitary adenomas stained positive for ACTH and expressed the T-pit transcription factor (**Figure 3**).

Day 3 after surgery, cortisol, ACTH, and 24h UFC levels had normalized, achieving complete biochemical remission. The patient’s pituitary function largely normalized, with a slight decrease in testosterone levels, and no subsequent replacement therapy was administered.

## Methods

### Search strategy and selection criteria

The online database Pubmed was searched using the keywords “double pituitary adenomas”, “bilateral pituitary adenomas” and “ concurrent pituitary adenomas”, with a publication date range from January 1990 to June 2023 and the article type was limited to case reports. After reviewing the titles and abstracts of the retrieved results, 28 articles were selected for further full-text assessment for inclusion. The following articles were excluded: (1) articles not published in English, (2) no surgical treatment was performed, (3) no hormonal abnormalities or related endocrine symptoms prior to surgery, (4) no mention of postoperative biochemical remission results in the text, (5) two tumors that were not concurrent, namely recurrent pituitary adenomas. Finally, 57 patients from 22 articles (7–28) met the inclusion criteria, adding 2 patients reported in the paper, a total of 59 patients with functional pituitary adenomas were included in the systematic review.

### Statistical analysis

Continuous variables were described as mean ± standard deviation (SD). Categorical variables were described as numbers and percentages. The statistical significance in a 2 × 2 table was evaluated using Pearson’s chi-squared test or Fisher’s exact test. In the multivariate analysis, multivariate logistic regression was used to identify the independent prognostic factors. Two-tailed tests were used, and P-value <0.05 was considered statistically significant. All statistical analyses were performed using SPSS version 26 (IBM Corp., Armonk, New York) and GraphPad Prism 9 (GraphPad Software, California, USA).

## Results

### Demographics

**Figure 4:**
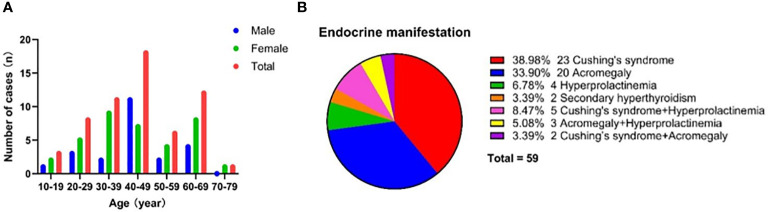
Demographic and clinical characteristics for patients with DPA. **(A)** Age and gander distribution for DPA patients. **(B)** Endocrine manifestations for DPA patients.

Among the 59 cases of functional DPA, 61.02% (n = 36) were female patients, demonstrating a slight female predominance. The mean ± SD age at diagnosis was 43.64 ± 14.42 years. Age data showed that the patients with functional DPA tended to be diagnosed in the fourth decade of life, followed by in the sixth and third decades (**Figure 4A**). The age at diagnosis showed no statistically significant difference between genders (43.61 ± 13.99 years for male vs 43.67 ± 14.88 years for female). The majority of functional DPA patients were sporadic cases, but 8.5% of patients (n = 5) had a hereditary background, including 3 cases of familial multiple endocrine neoplasia type 1 (MEN 1) and 2 cases of familial pituitary adenoma unrelated to MEN 1.

### Clinical manifestations

The endocrine manifestations of functional DPA patients are summarized in **Figure 4B**. The most prevalent clinical manifestation was Cushing’s syndrome (n = 23, 38.98%), followed by acromegaly (n = 20, 33.90%). Furthermore, only a minority of patients presented with hyperprolactinemia (n = 4, 6.78%) or secondary hyperthyroidism (n = 2, 3.39%). It was worth noting that 16.95% of patients (n = 10) had two types of endocrine symptoms simultaneously, which may be due to the fact that both double pituitary adenomas had secreting function or one of the adenomas was a plurihormonal adenoma. Hyperprolactinemia tended to manifest as an incidental symptom coexisting with Cushing’s syndrome or acromegaly.

### Diagnosis

The diagnostic methods and detection rates of DPA are shown in **Table 2**. Twenty-one cases of DPA were diagnosed by preoperative MRI, with an total detection rate of 35.6%. The use of higher field strength MRI was beneficial in improving the DPA detection rate. 3.0T MRI had a detection rate of 50.0%, which was significantly higher than that of 1.5T MRI of 22.6%, and the difference reached a statistically significant level (P = 0.034). The DPA detection rate of surgical exploration was 47.5%, which was slightly higher than that of preoperative MRI, indicating that some MRI-negative adenomas were detected under direct surgical visualization. No statistically significant difference was seen when comparing the detection rates of the two surgical approaches, using endoscope and microscope (P = 0.734).

**Table 2:** Diagnostic methods and detection rates for DPA.

Diagnosis methods	Total	Single lesion visible	Double lesions visible	P-value
MRI				
1.5T	31	24 (77.4%)	7 (22.6%)	0.034*
3.0T	28	14 (50.0%)	14 (50.0%)	
Total	59	38 (64.4%)	21 (35.6%)	
Surgery				
Microscope	10	6 (60.0%)	4 (40.0%)	0.734
Endoscope	49	25 (51.0%)	24 (49.0%)	
Total	59	31 (52.5%)	38 (47.5%)	

*P < 0.05.

Of all 59 cases of DPA, the separated type accounted for 46 cases (78.0%) and the contiguous type accounted for 13 cases (22.0%). It is noteworthy that because the contiguous type DPA presented as a whole mass in appearance, neither MRI examination nor surgical exploration could detect the double lesions. All 13 cases of contiguous type DPA were definitively diagnosed on postoperative pathological examination.

### Tumor characteristics

**Figure 5:**
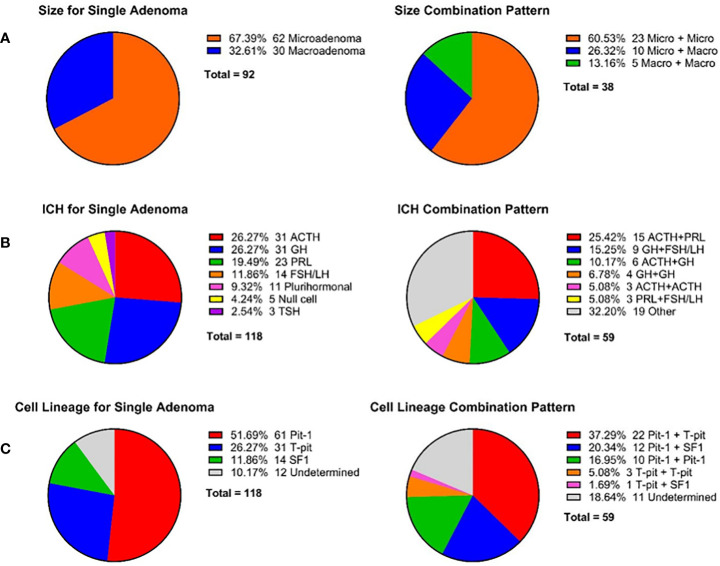
Tumor characteristics for patients with DPA. **(A)** Tumor size for single adenoma and combination pattern for each DPA patient. Tumor size was assessed through MRI, surgery, or pathological examination. 38 cases reported sizes for both double pituitary adenomas, while another 16 cases reported size for only one of the double pituitary adenomas. **(B)** Pituitary hormone immunohistochemical staining of single adenoma and combination patterns for each DPA patients. ICH, immunohistochemistry. **(C)** Cell lineage for single adenoma and combination pattern for each DPA patient.

**Figure 5** summarizes the tumor size and immunohistochemical characteristics of DPA. Among the 92 adenomas for which tumor size data were available in case reports, approximately two-thirds were microadenomas (n = 62, 67.39%), while about one-third were macroadenomas (n = 30, 32.61%). There are currently no reported cases of giant adenomas. The most common tumor size combination pattern for DPA is Micro+Micro (n = 23, 60.53%), followed by Micro + Macro (n =10, 26.32%), while Macro+Macro is the least common (n = 5, 13.16%).

In terms of immunohistochemistry, among the 118 adenomas in 59 patients, the most common were ACTH-immunopositive (n = 31, 26.27%) and GH-immunopositive tumors (n = 31, 26.27%). The majority of these adenomas exhibited secretion activity (29/31 for ACTH and 28/31 for GH). PRL-immunopositive and FSH/LH-immunopositive adenomas accounted for 19.49% (n = 23) and 11.86% (n = 14), respectively, although they tended to be silent, with only eight PRL adenomas and one FSH/LH adenoma having secretory function. Notably, a proportion of DPA patients had plurihormonal adenomas (n = 11, 18.6%), the most common of which was GH-PRL mixed adenomas (n =4). The most frequent immunohistochemical combination patterns were ACTH+PRL (n = 15, 25.42%) and GH+LH/FSH (n = 9, 15.25%), typically associated with secretion in the former and silence in the latter.

According to the WHO 2022 classification of pituitary neuroendocrine tumors, they are categorized into three cell lineages: Pit-1 (GH-PRL-TSH adenomas), T-pit (ACTH adenomas), and SF1 (FSH-LH adenomas), accounting for 51.69% (n = 61), 26.27% (n = 31) and 11.86% (n = 14), respectively. The common combination patterns include Pit-1 + T-pit (n = 22, 37.29%), Pit-1 + SF1 (n = 12, 20.34%), and double Pit-1 (n =10, 16.95%).

### Clinical outcomes and prognostic factors

In the cohort of 59 DPA patients, the postoperative biochemical complete remission (CR) rate was 74.6% (n = 44), while 25.4 of patients (n = 15) achieved only biochemical partial remission (PR) or no change (NC), requiring ongoing medication or secondary surgery. Gender, hereditary PA, clinical features, MRI detection and field strength, surgical exploration and approach, tumor type, and tumor size were assessed as prognostic factors for their correlation with postoperative biochemical remission (**Table 3**). Immunohistochemistry and cell lineage were not included in the analysis due to the extensive variety of categories. In the univariate analysis, it was observed that female patients (P = 0.030) and patients presenting with Cushing’s syndrome (P = 0.042) had a worse prognosis, while double lesions were detected by surgical exploration exhibited a significantly better prognosis (P = 0.014). Contiguous type DPA also showed a tendency toward a more favorable prognosis, although statistical significance was not reached (P = 0.091). The four prognostic factors (gender, Cushing’s syndrome, surgical exploration, and tumor type) with a P-value of less than 0.1 in the univariate analysis were included in binary logistic regression. The results of the multivariate analysis demonstrated that the contiguous type (OR = 0.06, P = 0.017) and surgical exploration (OR = 0.08, P = 0.003) were independent protective factors for the prognosis of DPA patients.

**Table 3:** Prognostic analysis of postoperative biochemical remission in patients with DPA.

	Total	CR	PR/NC	Univariate	Multivariate	
				P-value	P-value	OR (95% CI)
**Gender**						
Male	23	21	2	0.030*	0.072	5.71 (0.86-38.06)
Female	36	23	13			
**Hereditary PA**						
No	54	39	15	0.315		
Yes	5	5	0			
**Cushing’s syndrome**						
No	29	25	4	0.042*	0.735	1.32 (0.26-6.71)
Yes	30	19	11			
**MRI detection**						
Double lesions visible	21	17	4	0.305		
Single lesion visible	38	27	11			
**MRI field strength**						
1.5T	31	24	7	0.409		
3.0T	28	20	8			
**Surgical exploration**						
Double lesions visible	28	25	3	0.014*	0.003**	0.08 (0.02-0.42)
Single lesion visible	31	19	12			
**Surgical approaches**						
Microscope	10	7	3	0.495		
Endoscope	49	37	12			
**Tumor type**						
Separated type	46	32	14	0.091^#^	0.017*	0.06 (0.01-0.59)
Contiguous type	13	12	1			
**Tumor size**						
Micro + Micro	23	16	7	0.618		
Micro + Macro	10	6	5			
Macro + Macro	5	4	1			

CR, complete remission; ICH, immunohistochemistry; NC, no change; PA, pituitary adenoma; PR, partial remission.

^#^ P < 0.1, * P < 0.05, ** P < 0.01.Bolded values represent a statistically significant p-value of <0.05.

## Discussion

DPA is a rare clinical condition, and its incidence is one of the current research concerns. The incidence of DPA from randomly selected autopsy material ranges from 7.0-10.5% of adenomatous pituitary (29–31). The incidence of DPA estimated from surgical specimens varies over a wide range, from 0.16-2.6% has been reported (13, 16, 32, 33). The prevalence of DPA was higher in the surgical cohort of patients with Cushing’s disease, with 3.3% of 660 patients having double lesions (9). Comparing incidence from autopsy and surgical cohorts, we suggest that DPAs are not actually as rare in pituitary adenomas as they are seen clinically, but that the majority of DPAs are small-sized and non-functioning subclinical cases, leading to a significant underestimation of DPA incidence in surgical cohorts.

In this study, the mean age at diagnosis of DPA was 43.64 years, with a peak age of the fourth decade of life and a male-to-female ratio of approximately 2:3, suggesting that the age-sex profile of DPA is similar to that of the common solitary pituitary adenoma (34). Previous studies have concluded that Cushing’s disease and acromegaly are the most common clinical manifestations of DPA (6, 32), and this paper reconfirms this finding. The most frequent immunohistochemical types of DPA have reached a preliminary consensus (ACTH- and GH-immunopositive for single adenoma, ACTH+PRL and GH+FSH/LH for combination pattern), as reported in both this study and previous literature (35). Systematic summarization of clinical DPA cases revealed that PRL-immunopositive tumors tend to be silent in the setting of DPA, the mechanism of which is currently unknown. In contrast to surgical series, studies based on autopsy material have suggested that PRL-immunopositivity is the most common immunohistochemical type (29, 36). Therefore, it is hypothesized that PRL-immunopositive tumors have a high incidence in DPA but are typically distributed as silent forms in subclinical cases. Regarding tumor size, the traditional view based on autopsy series is that almost all DPAs are microadenomas (29, 37). However, this study found that macroadenomas accounted for a significant proportion (32.6%), which reflects the difference between subclinical and clinical cases.

In clinical practice, it is essential for clinicians to enhance their awareness of this rare condition — DPA, be vigilant for clinical scenarios suggestive of DPA, and identify patients at high risk for hidden dual lesions. The presence of two distinct endocrine manifestations or markedly elevated levels of two pituitary hormones is a crucial clue indicating DPA. Although hyperprolactinemia often manifests as an incidental symptom coexisting with Cushing’s syndrome or acromegaly, it must be distinguished from the pituitary stalk effect, with serum prolactin levels above 200 ng/mL supporting a primary prolactinoma diagnosis (38). Moreover, the elevation of two hormones may also result from a plurihormonal adenoma that simultaneously secretes both hormones (25), which requires confirmation upon pathological examination. Case reports have shown that bromocriptine treatment for prolactinomas may yield incomplete responses, where serum prolactin levels decreased but MRI did not show the expected degree of shrinkage of the tumor (39, 40). This phenomenon may be attributed to DPA, where the tumor visible on MRI consists partly or entirely of non-prolactinoma components, leading to poor response to medical treatment. In Cushing’s disease, if BIPSS suggests a dominant side inconsistent with the tumor’s MRI location, further evaluation is needed to avoid missing a hidden second lesion. In these specific clinical scenarios, clinicians should promptly consider the possibility of DPA and perform MRI reading and intraoperative exploration with extra care to achieve an early diagnosis.

The conventional perspective holds that the preoperative identification of DPA through imaging is challenging (8, 9, 18, 21), and this can be attributed to several factors: (1) Most DPAs typically include at least one small-sized adenoma (6), and (2) Cushing’s disease is the most common clinical manifestation of DPA, but MRI-negative tumors are more common among ACTH-secreting tumors (41, 42). Currently, the standard imaging method for diagnosing pituitary adenomas is dynamic contrast-enhanced pituitary MRI with thin-layer scanning and 3.0T field strength. In this study, we found 3.0T MRI significantly improved the detection rate of DPA compared with 1.5T MRI. This suggests that preoperative imaging detection of DPA depends on the development and clinical application of advanced imaging technologies. In addition to higher field strength, novel sequences and functional imaging fusion are also promising ways to improve image quality. Previous literature has demonstrated that using spoiled gradient recalled acquisition (SPGR) sequences can enhance the spatial resolution and sensitivity of MRI for the detection of ACTH-secreting adenomas, albeit with a mild loss of specificity (21, 43). What’s more (11).C-MET-PET/3.0T MRI showed higher sensitivity in the detection of ACTH-secreting pituitary microadenomas compared to traditional and dynamic MRI, which is an emerging imaging technology for future DPA diagnosis (44, 45).

Despite the use of 3.0T MRI, half of the DPA cases are not diagnosed on preoperative MRI. Therefore, preoperative imaging results should not be blindly trusted, and comprehensive intraoperative pituitary exploration is an important prerequisite for complete resection of double lesions. The importance for intraoperative exploration is further emphasized by the present study, which demonstrated double lesions detected by intraoperative exploration was an independent prognostic factor for postoperative biochemical remission. Preoperative MRI should be used as an imaging tool to guide intraoperative exploration by providing location information, but intraoperative exploration under direct visualization is an indispensable step regardless of how many lesions are detected by MRI. As mentioned above, elevated levels of two pituitary hormones, poor response of bromocriptine treatment, inconsistency between the dominant side of BIPSS and MRI, or pituitary abnormalities of undetermined significance on imaging should be considered as high-risk features for DPA. For high-risk patients, intraoperative exploration should be extra careful to exclude lesion missed. Pituitary surface exploration can largely reduce the omission of second lesions, as more than 50% of small tumors are distributed on the pituitary surface (46). Pituitary incisional exploration requires a patient-specific weighing of the importance of preserving pituitary function and avoiding missed lesions. The use of intraoperative pituitary imaging is beneficial for tumor identification and complete resection. Intraoperative ultrasound has been shown to detect some MRI-negative pituitary adenomas (47, 48), and the use of intraoperative MRI can help to identify residual tumors and increase the rate of complete resection (49, 50). For patients with Cushing’s disease, as MRI-negative tumors are more common among ACTH-secreting tumors, several cases of biochemical non-remission after surgery due to failing to remove the causative tumor have been reported (9, 12, 21). In this study, univariate analysis revealed significantly worse biochemical remission in patients with Cushing’s disease (P = 0.042), although no statistical significance was observed in multivariate analysis. Therefore, confirming the complete removal of tumors during surgery is particularly important for patients with Cushing’s disease. Determination of the attenuation of ACTH concentration after tumor resection by cavernous sinus blood sampling (51, 52) and quantitative analysis of ACTH in tumor tissues (53) are promising methods for confirming complete resection of ACTH-secreting tumors, but they are still not widely used in clinical practice.

Contiguous type DPA cannot be detected through preoperative MRI or intraoperative exploration, even if the images are retrospectively reviewed by experienced radiologists and the pituitary gland is carefully explored by experienced neurosurgeons (40). However, in this study, the rate of postoperative biochemical CR reached 92.3% in contiguous type DPA, which was an independent protective factor for postoperative biochemical remission. Contiguous type DPA is usually completely removed as a single mass during surgery, and the diagnosis can only be confirmed by the identification of two cell populations with distinct immunohistochemiscal features on postoperative pathological examination. Therefore, theoretically, there is no possibility of surgical failure due to missing one of the hormone-secreting tumors, which provides a reasonable explanation for its significantly better prognosis. Despite the favorable prognosis of contiguous type DPA compared to separated type DPA, it is not possible to accurately differentiate between these two tumor types until postoperative pathology is obtained. Therefore, there is no difference in clinical management between the two types of DPA, and careful MRI reading and comprehensive intraoperative exploration are not negligible for both tumor types.

This study is the first systematic review to analyze the prognostic factors affecting postoperative biochemical remission in patients with DPA and to provide meaningful insights into the clinical practice of this rare clinical condition of DPA. However, there are several limitations to this study. This is a retrospective study, whose data were derived from cases reported by our institution and collected from previous literature. Therefore, selection bias, missing data, and inaccurate information are unavoidable. Restricted by the very low incidence of DPA and the limited number of available case reports, the small sample size of this study was insufficient to produce more reliable and accurate results. The clinical information provided by the included case reports in this study exhibits significant heterogeneity. For example, tumor size was measured by different methods, including MRI, surgery, or pathology. Therefore, this article provides only preliminary experience in the prognosis and clinical management of DPA, and further research involving a larger number of patients is anticipated in the future.

## Conclusion

The peak age of the occurrence of DPA is in the forties, with a slight female predominance. The most common clinical manifestations are Cushing’s disease and acromegaly. The most common immunohistochemical types are ACTH- and GH-immunopositive tumors, often occurring as ACTH+PRL and GH+FSH/LH combination patterns. PRL-immunopositive tumors tend to be silent in the setting of DPA. Double microadenomas are the most common tumor size combination, but macroadenomas+microadenomas are more frequent than previously recognized. 3.0T MRI significantly improved the detected rate of DPA compared with 1.5T MRI, and more novel imaging technology (eg. SPGR sequence, 11C-MET-PET/3.0T MRI) is promising. Double lesions detected by surgical exploration and contiguous type tumor are independent protective factors for postoperative biochemical remission in patients with DPA. Comprehensive intraoperative exploration is indispensable to avoid missing the second lesion.

## Data availability statement

The original contributions presented in the study are included in the article/supplementary material. Further inquiries can be directed to the corresponding authors.

## Author contributions

YZ: Conceptualization, Data curation, Formal Analysis, Funding acquisition, Methodology, Writing – original draft, Writing – review & editing. XG: Formal Analysis, Funding acquisition, Writing – original draft, Writing – review & editing. JP: Methodology, Writing – review & editing. JL: Methodology, Writing – review & editing. ZY: Resources, Writing – review & editing. HZ: Resources, Writing – review & editing. LL: Resources, Writing – review & editing. HP: Methodology, Writing – review & editing. KD: Conceptualization, Supervision, Writing – review & editing. YY: Conceptualization, Supervision, Writing – review & editing.
